# Supplementary material for: Synthesis, Characterization, and Biological Evaluation of Some Novel Pyrazolo[5,1-b]thiazole Derivatives as Potential Antimicrobial and Anticancer Agents
Source: Molecules. 2021 Sep 4;26(17):5383. doi: 10.3390/molecules26175383 (PMC8434505; doi:10.3390/molecules26175383)
Supplement: Supplementary file 1 [file molecules-26-05383-s001.zip › molecules-1340004-supplementary.pdf]

Synthesis, Characterization, and Biological Evaluation of some Novel pyrazolo[5,1-*b*]thiazole Derivatives as Potential Antimicrobial and Anticancer Agents

Abdulrhman Alsayari [1], Abdullatif Bin Muhsinah [1], Yahya I. Asiri [2], Faiz A. Al-aizari [3,4], Nabila A. Kheder [5], Zainab M. Almarhoon [3], Hazem A. Ghabbour [6] and Yahia N. Mabkhot [7,*]

Experiments.

Chemistry

Materials and equipment

All chemicals used in this work were obtained from commercial sources and were used as received without further purification. Melting points (uncorrected) were measured with a Gallenkamp melting point apparatus. Infrared (IR) spectra were recorded as KBr discs on a Perkin Elmer FT-IR 1000 spectrometer. The ^{1}H and ^{13}C NMR spectra were obtained with the aid of a Varian Mercury Jeol-400 NMR spectrometer and are reported in ppm (δ) relative to tetramethylsilane as an internal standard and with $CDCl_3$ as a solvent. Chemical shifts are expressed in δ units; coupling constants (J-values) for ^{1}H–^{1}H coupling are given in Hertz. Abbreviations for multiplicity are as follows: s (singlet), d (doublet), t (triplet), q (quadruplet), and m (multiplet). Mass spectra were recorded on a Shimadzu GCMS-QP 1000 EX mass spectrometer at 70 eV. Elemental analyses were carried out on an Elemental Vario EL analyzer. The single-crystal X-ray diffraction measurements were performed on a SMART APEX II CCD diffractometer (Bruker AXS Advanced X-ray Solutions GmbH, Karlsruhe, Germany). The final refinement was carried out by full-matrix least-squares techniques with anisotropic thermal data for nonhydrogen atoms on *F*. **CCDC 2075096** contains the supplementary

crystallographic data for this paper and can be obtained free of charge from the Cambridge Crystallographic Data Centre via www.ccdc.cam.ac.uk/data_request/cif.

Biological tests.

Method of evaluation of the cytotoxic effects of the synthesized compounds

Mammalian cell lines: HepG-2 cells (human Hepatocellular carcinoma) and HCT-116 cells (colon carcinoma) were obtained from the VACSERA Tissue Culture Unit. Chemicals used: Dimethyl sulfoxide (DMSO), crystal violet and trypan blue dye were purchased from Sigma (St. Louis, Mo., USA). Fetal bovine serum, DMEM, RPMI-1640, HEPES buffer solution, L-glutamine, gentamycin and 0.25% Trypsin-EDTA were purchased from Lonza. Crystal violet stain (1%) was composed of 0.5% (w/v) crystal violet and 50% methanol, then made up to volume with ddH_2O and filtered through a Whatmann No.1 filter paper.

Cell line Propagation:

The cells were propagated in Dulbecco's modified Eagle's medium (DMEM) supplemented with 10% heat-inactivated fetal bovine serum, 1% L-glutamine, HEPES buffer and 50μg/ml gentamycin. All cells were maintained at 37ºC in a humidified atmosphere with 5% CO_2 and were subcultured two times a week.

Cytotoxicity evaluation using viability assay: For the cytotoxicity assay, the cells were seeded in a 96-well plate at a cell concentration of 1×10^4 cells per well in 100 μl of growth medium. Fresh medium containing different concentrations of the test sample was added after 24 h of seeding. Serial two-fold dilutions of the tested chemical compound were added to confluent cell monolayers dispensed into 96-well, flat-bottomed microtiter plates (Falcon, NJ, USA) using a multichannel pipette.

The microtiter plates were incubated at 37ºC in a humidified incubator with 5% CO_2 for a period of 24 h. Three wells were used for each concentration of the test sample. Control cells were incubated without the test sample and with or without DMSO. The small percentage of DMSO present in the wells (maximal 0.1%) was found not to affect the experiment. After incubation of the cells at 37°C for 24 h, the viable cells yield was determined by a colorimetric method [52] In brief, after the end of the incubation period, media were aspirated and the crystal violet solution (1%) was added to each well for at least 30 minutes. The stain was removed, and the plates were rinsed using tap water until all excess stain was removed. Glacial acetic acid (30%) was then added to all wells and mixed thoroughly, and then the absorbance of the plates were measured after being gently shaken on a microplate reader (TECAN, Inc.), using a test wavelength of 490 nm. All results were corrected for background absorbance detected in wells without added stain. The treated samples were compared with the cell control in the absence of the tested compounds. All experiments were carried out in triplicate. The cell cytotoxic effect of each tested compound was calculated. The optical density was measured with the microplate reader (SunRise, TECAN, Inc, USA) to determine the number of viable cells, and the percentage of viability was calculated as [(ODt/ODc)]x100%, where ODt is the mean optical density of wells treated with the tested sample and ODc is the mean optical density of untreated cells. The relation between surviving cells and drug concentration was plotted to determine the survival curve of each tumor cell line after treatment with the specified compound. The 50% inhibitory concentration (IC_{50}), the concentration required to cause toxic effects in 50% of intact cells, was estimated from graphic plots of the dose–response curve for each concentration using Graphpad Prism software (San Diego, CA. USA).

Method of antimicrobial evaluation of the synthesized pyrazolo[5,1-*b*]thiazole derivatives

All microbial strains were provided from culture collection of the Regional Center for Mycology and Biotechnology (RCMB), Al-Azhar University, Cairo, Egypt. The antimicrobial activity was investigated on a newly synthesized compound in order to increase the selectivity of these derivatives towards test microorganisms using the agar diffusion method using Mueller–Hinton agar medium for bacteria and Sabouraud's agar medium for fungi. Briefly, 100 µl of the test bacteria/fungi were grown in 10 mL of fresh media until they reached a count of approximately10^8 cells/ml for bacteria or 10^5 cells/mL for fungi. All the newly synthesized compounds were weighed and dissolved in dimethyl sulfoxide to prepare extract stock solution. An amount of 100 µL of each sample at 5 mg/mL was added to each well (10 mm diameter holes cut in the agar gel). The plates were incubated for 24–48 h at 37 °C (for bacteria and yeast) and for 48 h at 28 °C (for filamentous fungi). After incubation, the microorganism's growth was observed. The resulting inhibition zone diameters were measured in millimeters and used as criterion for the antimicrobial activity. The size of this clear zone is proportional to the inhibitory action of the compound under investigation. DMSO was used to dissolve the tested compounds and thus used as solvent control, and showed no inhibition zones, confirming that it has no influence on the growth of the tested microorganisms. Positive controls were also performed using Ampicillin and Gentamycin as standard antibacterial drugs and amphotericin B as the standard antifungal drug [55].

Spectra

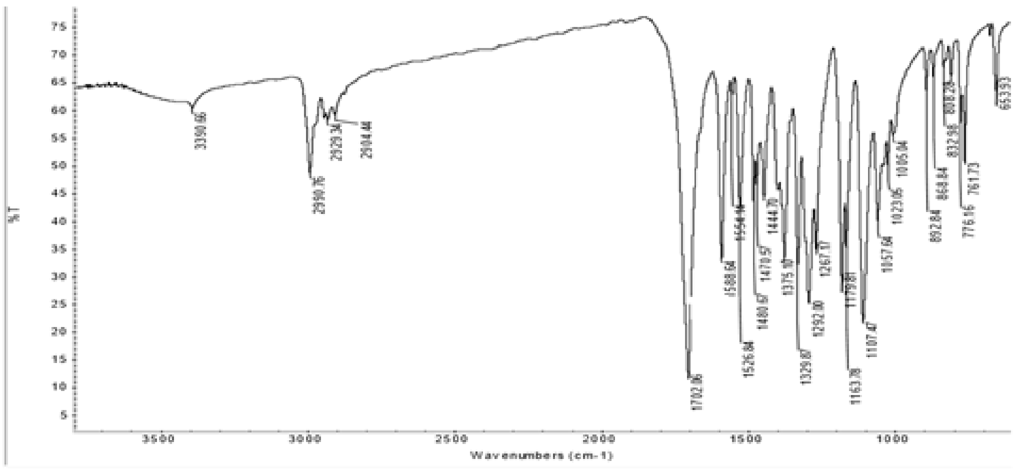

Figure S1. IR spectrum of compound **1.**

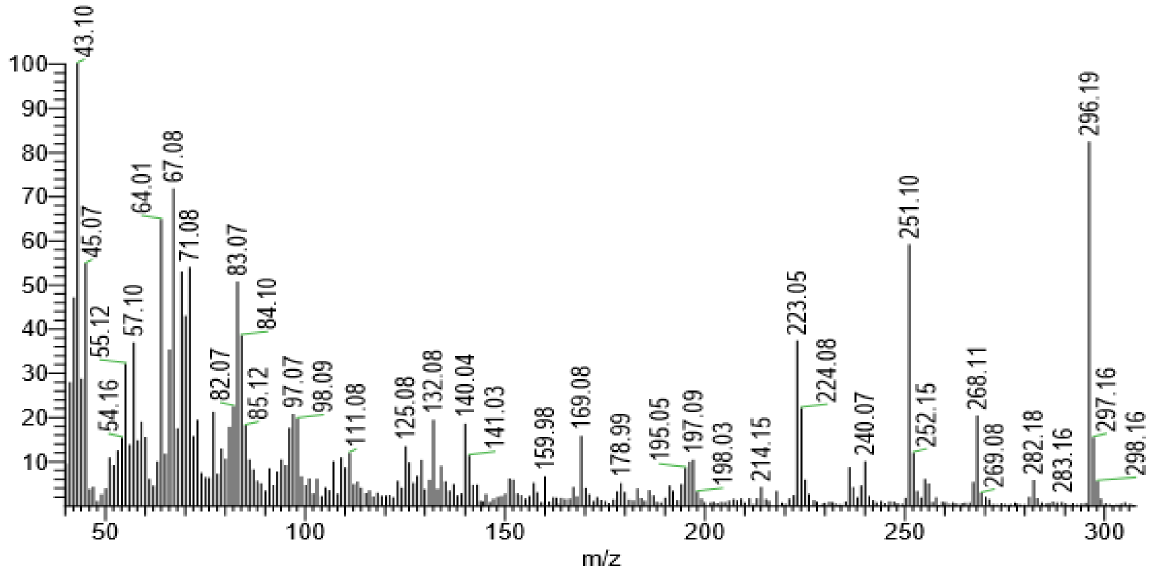

Figure S2. Mass spectrum of compound **1.**

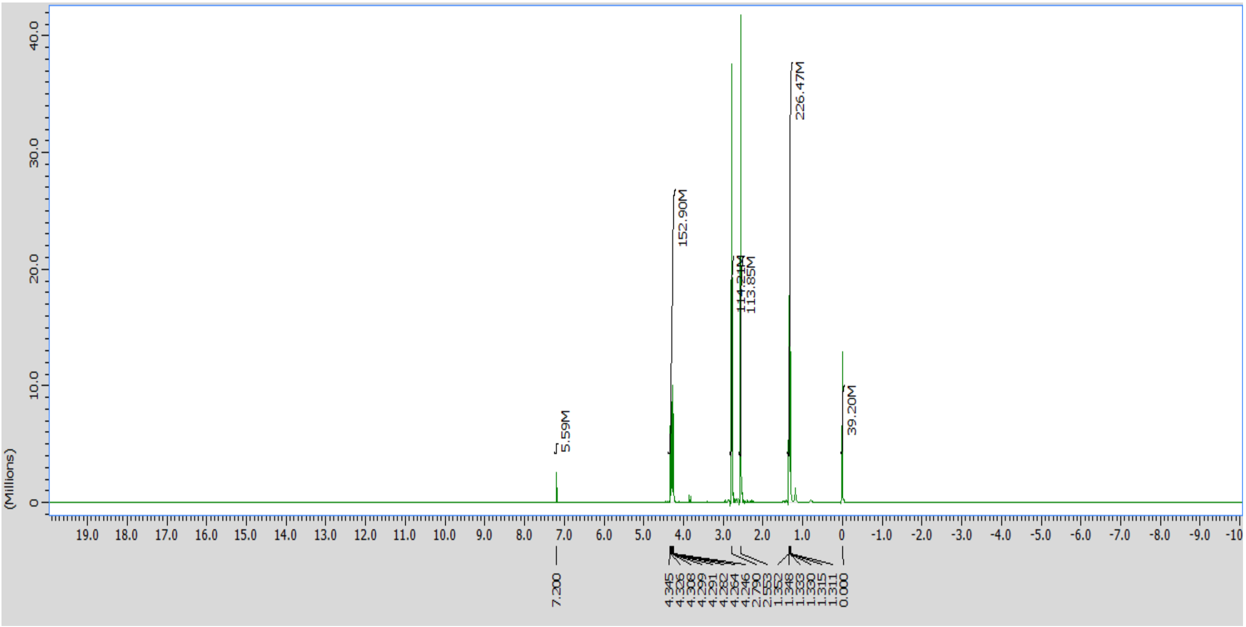

Figure S3. ¹H NMR spectrum of pyrazolo[5,1-b]thiazole derivative **1.**

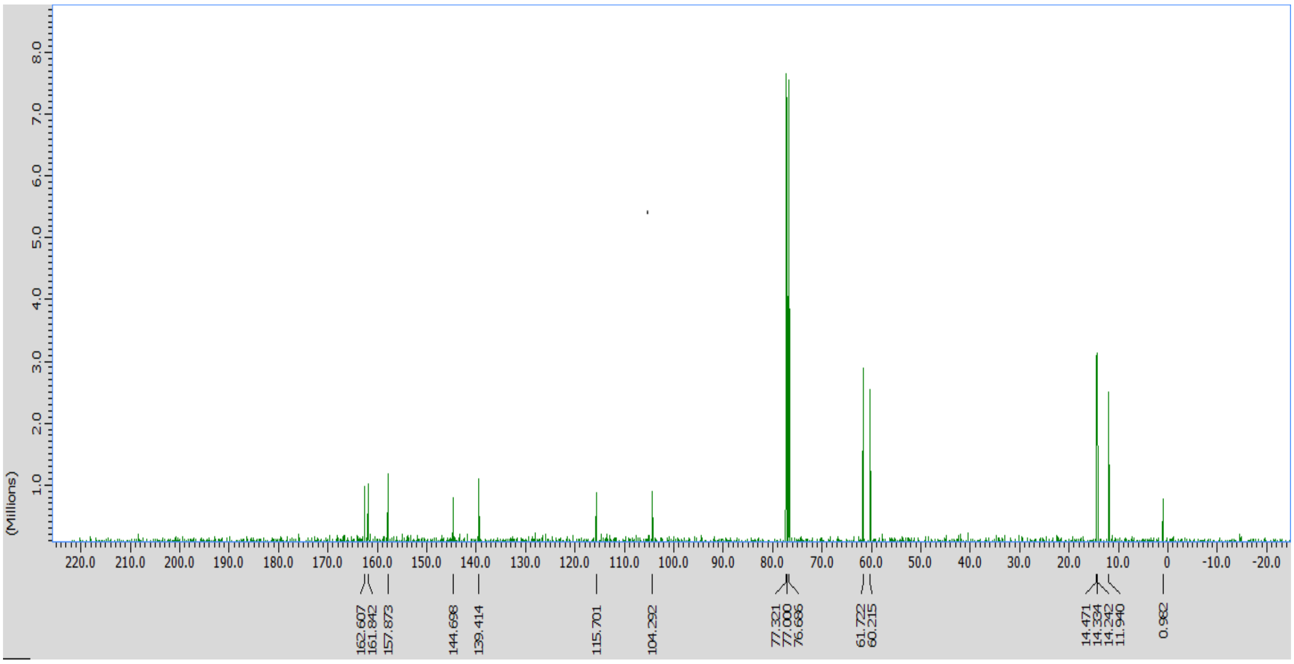

Figure S4. ¹³C NMR of compound **1.**

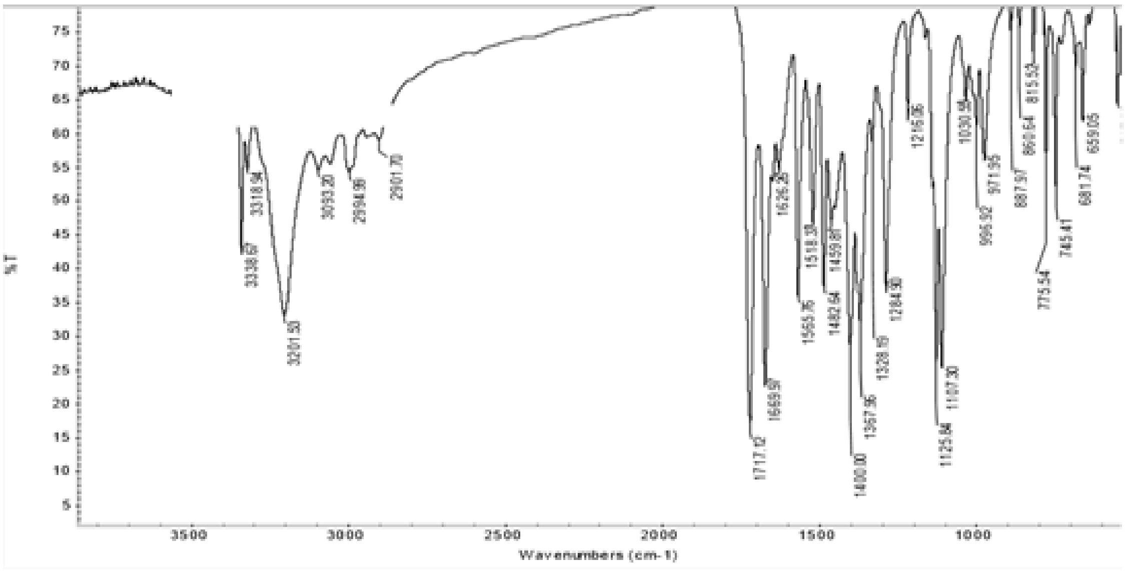

Figure S5. IR spectrum of hydrazide **2.**

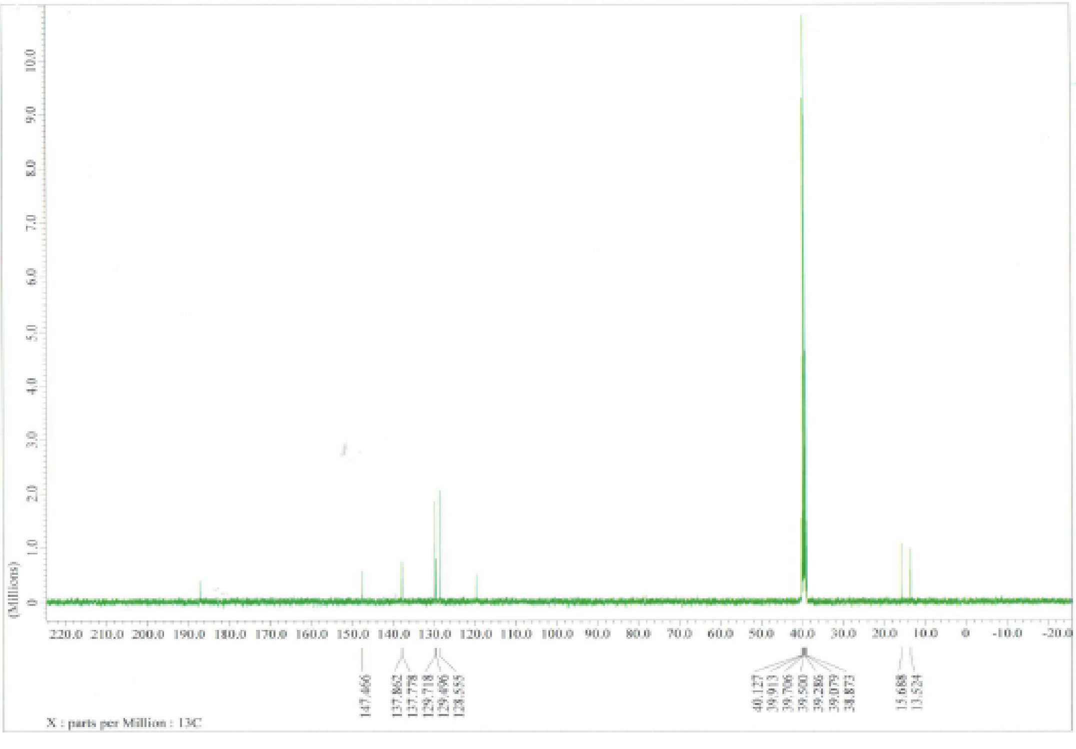

Figure S6. ^{13}C NMR of target compound **2.**

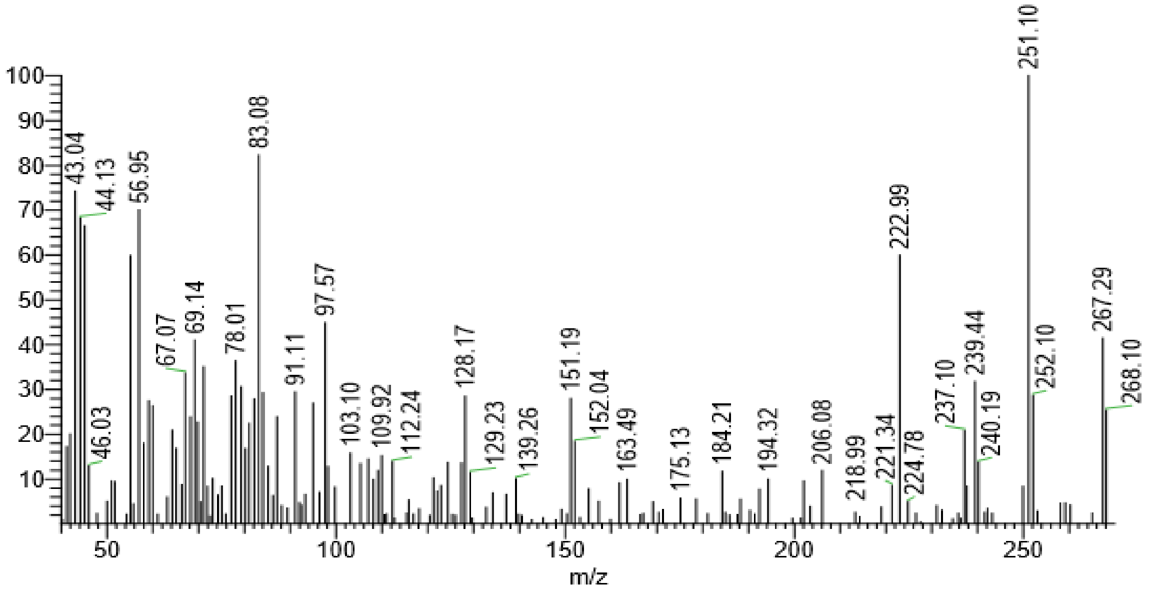

Figure S7. Mass spectrum of hydrazide **2.**

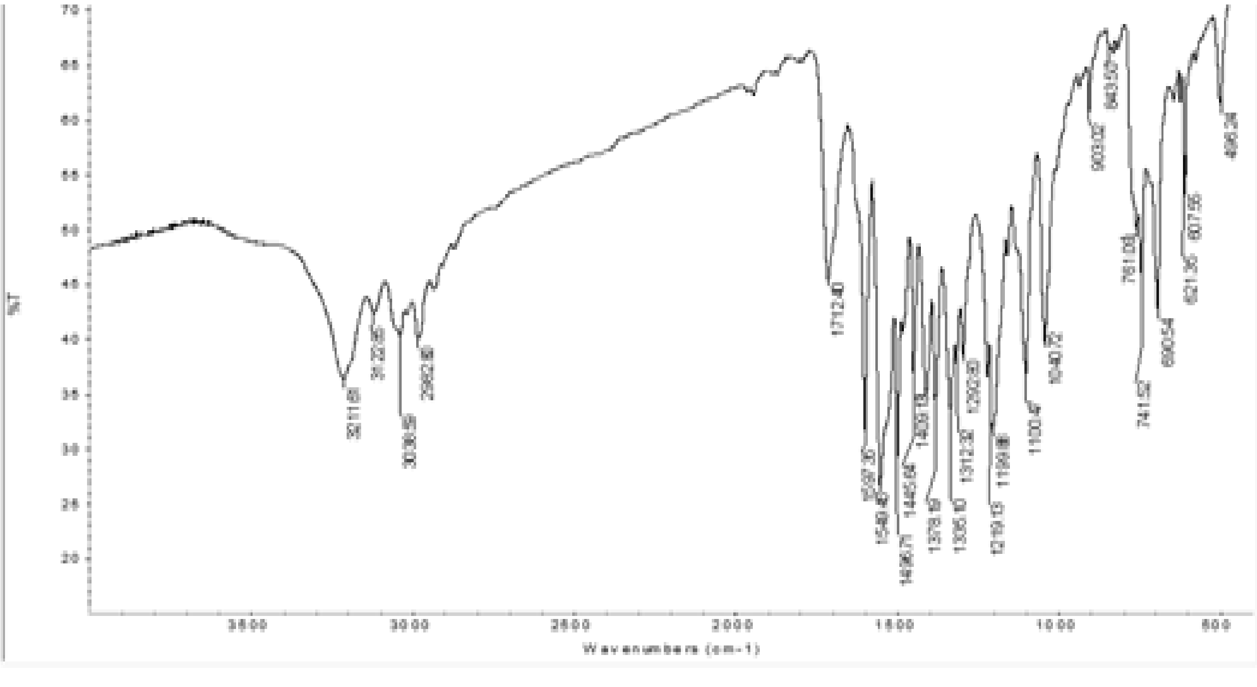

Figure S8. IR spectrum of compound **4.**

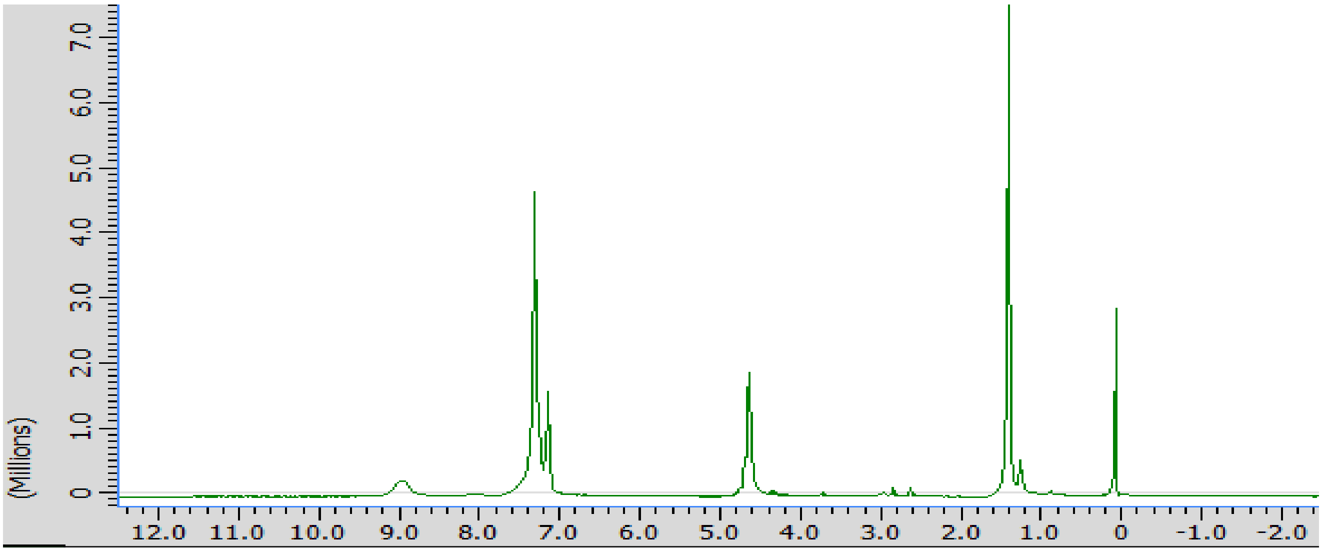

Figure S9. ^{1}H NMR spectrum of **4.**

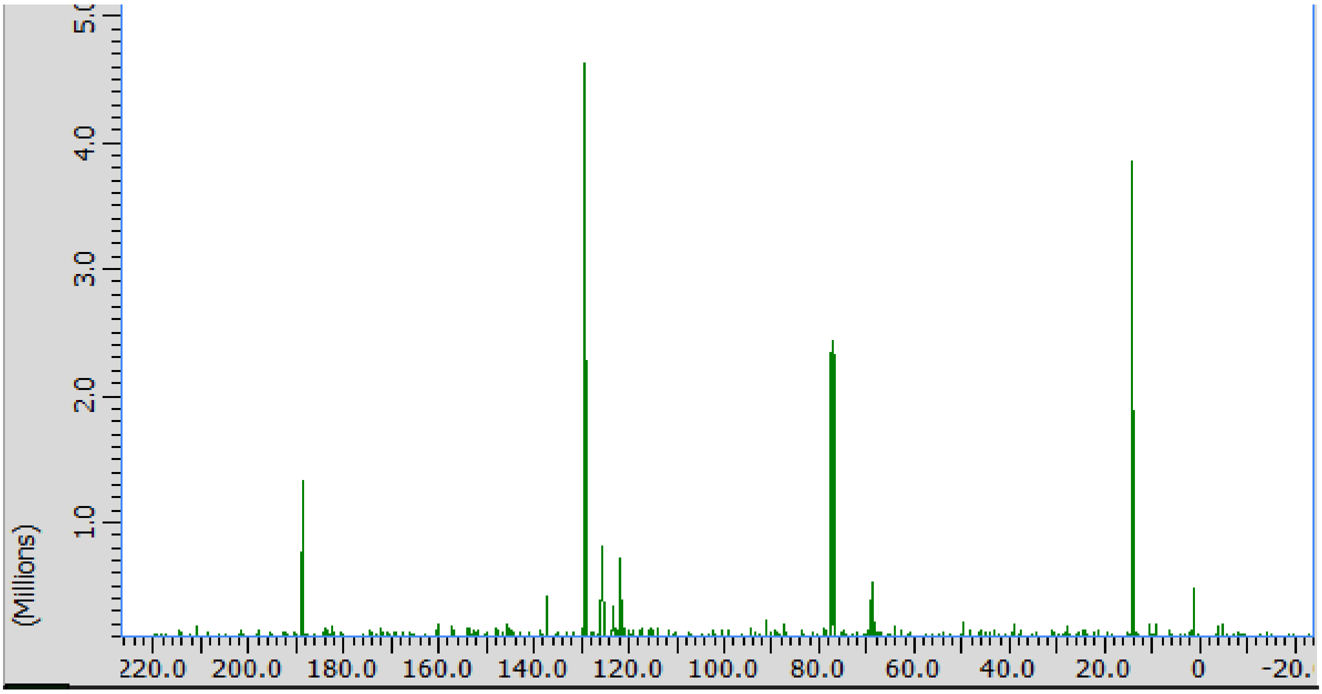

Figure S10. ^{13}C NMR spectrum of compound **4.**

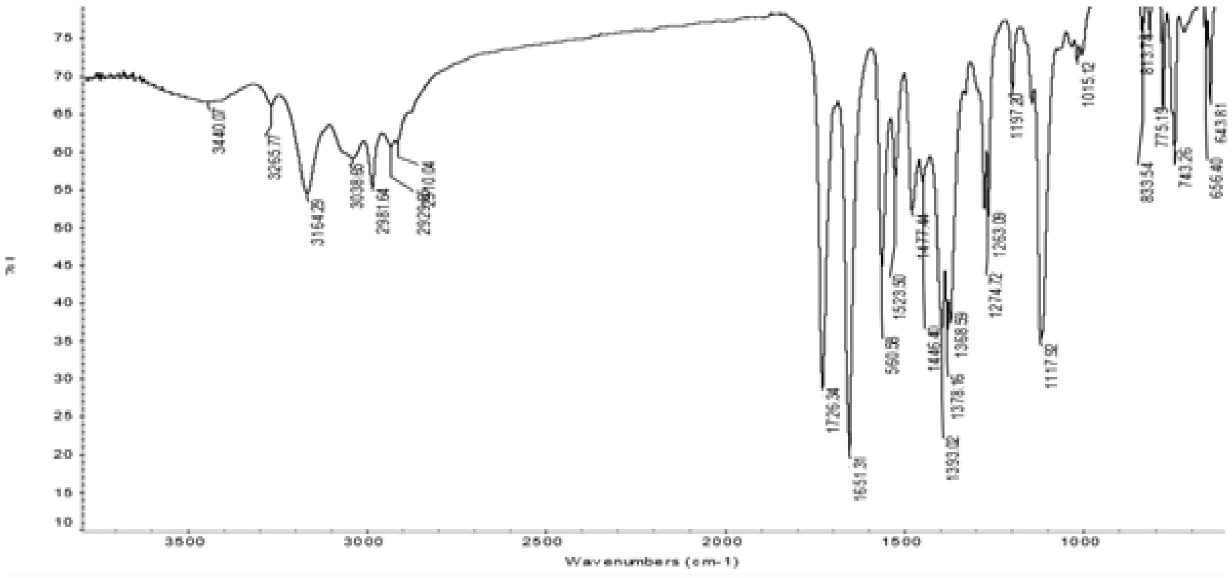

Figure S11. IR spectrum of compound **7.**

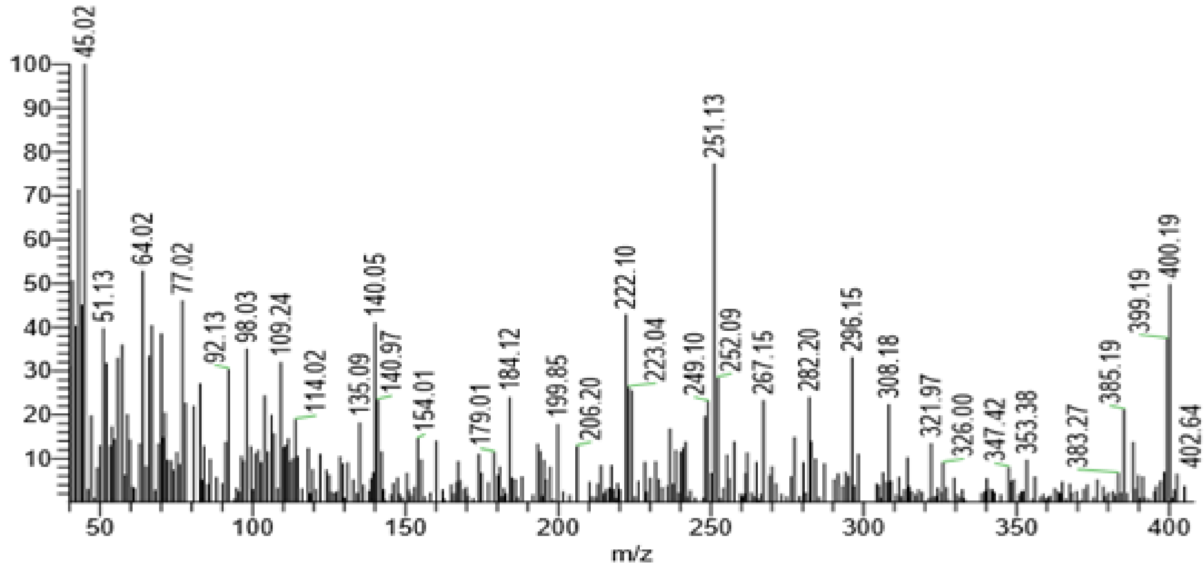

Figure S12. Mass spectrum of compound **7.**

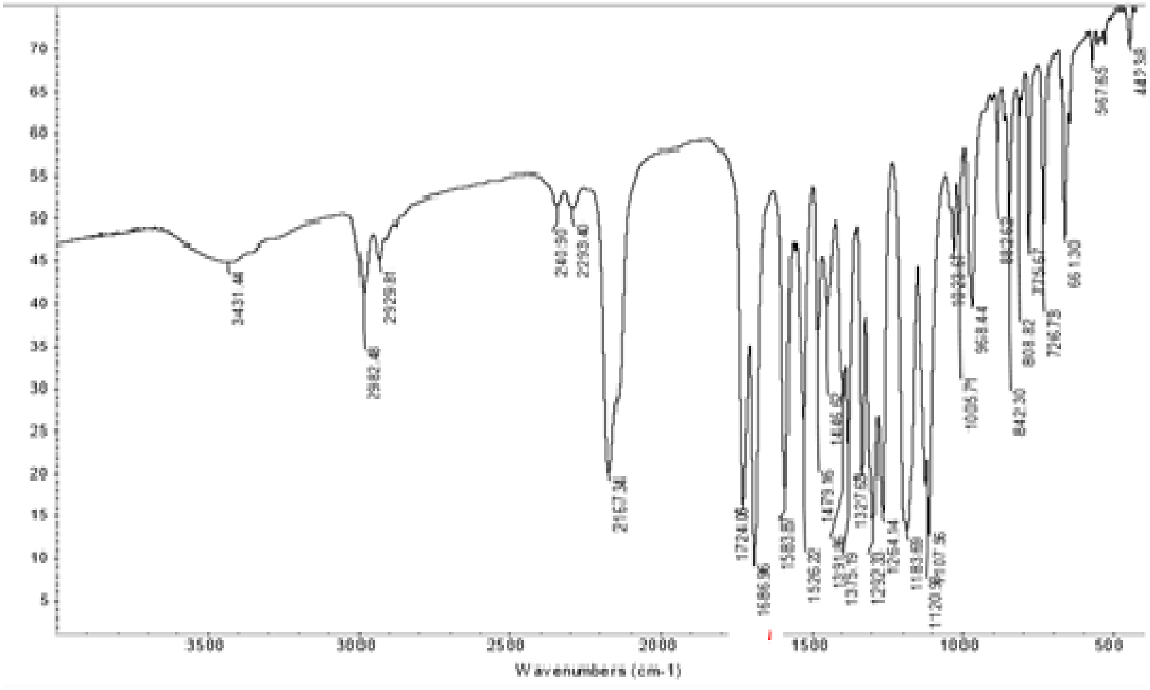

Figure S13. IR spectrum of azide **8.**

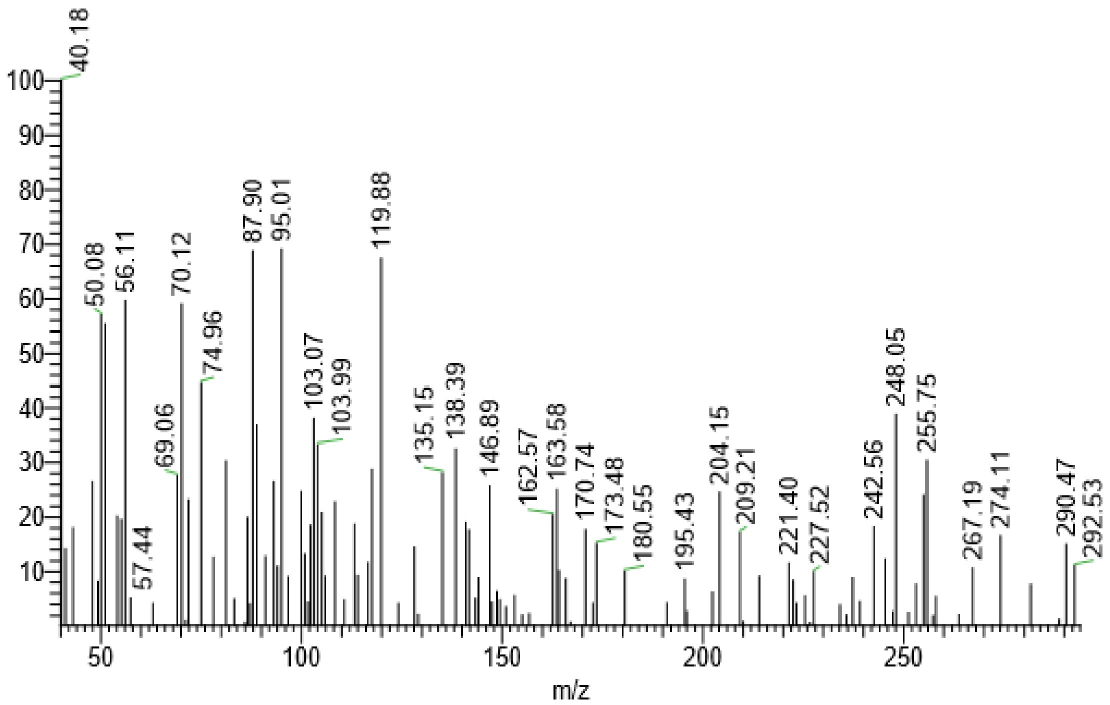

Figure S14. Mass spectrum of compound **8.**
